# Treatment-Free Outcomes Following Surgery for IBD: A Nationwide Cohort Study

**DOI:** 10.1111/apt.70432

**Published:** 2025-10-21

**Authors:** Adam S. Faye, Jordan Axelrad, Jiangwei Sun, Jonas Halfvarsson, SWIBREG Study Group, Par Myrelid, Jonas Söderling, Ola Olén, Jonas F. Ludvigsson

**Affiliations:** 1 Division of Gastroenterology, Department of Medicine, NYU School of Medicine, Inflammatory Bowel Disease Center at NYU Langone Health, New York, New York, USA; 2 Department of Medical Epidemiology and Biostatistics, Karolinska Institutet, Stockholm, Sweden; 3 Department of Gastroenterology, Faculty of Medicine and Health, Örebro University, Örebro, Sweden; 4 Department of Surgery, County Council of Östergötland and Department of Clinical and Experimental Medicine, Linköping University, Linköping, Sweden; 5 Division of Clinical Epidemiology, Department of Medicine Solna, Karolinska Institutet, Stockholm, Sweden; 6 Sachs’ Children and Youth Hospital, Stockholm South General Hospital, Stockholm, Sweden; 7 Department of Pediatrics, Örebro University Hospital, Örebro, Sweden; 8 Division of Digestive and Liver Disease, Department of Medicine, Columbia University Medical Center, New York, New York, USA

**Keywords:** Crohn’s disease, inflammatory bowel disease, surgery, ulcerative colitis

## Abstract

**Background:**

Surgery in select individuals with inflammatory bowel disease (IBD) may obviate the need for future IBD-related treatment.

**Aims:**

To characterise individuals who remain treatment-free during the first 5 years after initial IBD-related surgery.

**Methods:**

We performed a nationwide cohort study using the Swedish National Patient Register and the ESPRESSO histopathology to identify individuals undergoing first IBD-related intestinal resection for Crohn’s disease (CD) or total colectomy for ulcerative colitis (UC) between 2007 and 2018. We calculated adjusted odds ratios (aORs) for the need for any IBD-related therapy within the first 5 years post surgery.

**Results:**

We included 1709 individuals with CD and 1010 with UC. At 5 years, 21.5% with CD and 42.4% with UC remained ‘treatment free’. Being ‘treatment free’ 5 years after surgery was more common among patients with CD who had longer preoperative disease duration and older adults with UC. It was less common among individuals with extraintestinal manifestations of disease (CD aOR 0.64, 95% CI 0.43–0.97; UC aOR 0.48, 95% CI 0.31–0.73) and patients with CD who had chronic obstructive pulmonary disease.

**Conclusions:**

Surgery obviated the need for future therapy in 22% of patients with CD and 42% with UC. Absence of extraintestinal manifestations, older age in UC, and longer disease duration and absence of chronic obstructive pulmonary disease in CD may highlight an opportunity for precision surgery to identify those most likely to achieve long-term benefit from surgical intervention.

## 1 | Introduction

Inflammatory bowel disease (IBD), comprising Crohn’s disease (CD) and ulcerative colitis (UC), is a chronic immune-mediated disease of the gastrointestinal tract affecting more than 6 million individuals globally [1]. While medical therapy, including biologics and small molecules, remains the mainstay of treatment, a substantial proportion of patients will eventually require surgical intervention [2]. Although surgery has traditionally been reserved for patients who fail medical management, emerging evidence suggests that earlier surgical intervention may improve IBD-related outcomes. It can reduce complications of ongoing inflammation, lower the need for corticosteroids, decrease the risk of hospitalisation, and may improve overall mortality, particularly among older adults [3–5].

For select individuals, surgery may also act as a definitive treatment, obviating the need for any future IBD-related therapy (including both postoperative prophylaxis and treatment for active disease) [6]. In a post hoc analysis of the LIR!C study, 22% of individuals who had surgical resection for ileocecal CD did not require additional IBD-related prophylaxis or treatment for the following 5 years; findings that have since been confirmed with real-world data [5, 7]. This may be particularly beneficial among older adults, as IBD-related medical therapies carry a higher risk of adverse side effects in this patient population [8]. Further, because the window for postoperative recurrence is inherently shorter later in life, surgical intervention has the potential to serve as a definitive treatment option for some older adults with IBD [9].

While previous data suggest that older adults, non-tobacco users, and those with a longer disease course may have a lower risk of immediate postoperative recurrence in CD, there is limited information on the characteristics of patients with both UC and CD who undergo disease-related resection and remain free of IBD therapy for an extended period [9–11]. Thus, to bridge this knowledge gap, we conducted a nationwide cohort study to investigate the clinical characteristics of individuals with both UC and CD who underwent surgery and did not require any further IBD-related treatment for the following 5 years. Additionally, since older adults are considered to have a lower risk of recurrence and may represent a group with high potential for benefit, we performed additional subgroup analyses to inform future strategies for optimising surgical management in this population.

## 2 | Methods

### 2.1 | Data Sources

In Sweden, universal healthcare ensures that all residents are included in national patient registers with minimal loss to follow-up. Using the National Patient Register (NPR), we captured information regarding demographics, procedural codes, as well as all inpatient and outpatient contacts and International Classification of Diseases (ICD) codes for all residents. The accuracy of this register has been validated in previous studies, demonstrating positive predictive values most often ranging from 80% to 95% [12, 13]. Additionally, each resident in Sweden is assigned a unique personal identity number, which allows for linkage between the NPR and the Epidemiology Strengthened by Histopathology Reports (ESPRESSO) cohort. The ESPRESSO cohort compiles histopathology reports from 28 pathology laboratories across Sweden, encompassing over 8 million gastrointestinal pathology reports with detailed information on location, morphology, and diagnostic interpretations [14]. To capture information on IBD medication use, data were obtained from the Prescription Drug Register, which records details such as prescription dates, medication names, and both prescribed and dispensed doses for all medications filled at Swedish pharmacies [15].

### 2.2 | Study Design and Population

Our study population centered around individuals undergoing their first IBD-related intestinal resection. We defined the presence of IBD as having either ≥ 2 ICD codes relating to IBD in the NPR or one ICD code relating to IBD in the NPR in addition to ≥ 1 relevant histopathology code from the nationwide ESPRESSO cohort (Table S1). This method has been previously validated with an accuracy of 93%–95% [13, 16, 17]. Additionally, individuals with undifferentiated IBD were excluded from the study.

In order to accurately capture our study cohort, we identified all incident cases of IBD beginning in 2007 (not fulfilling the definition for IBD prior to 2007). This allowed us to exclude prevalent cases of IBD in which a prior intestinal resection may have occurred (hospital-based outpatient data were included in the NPR beginning in 2001, allowing for at least six prior years of complete data). Incident cases of IBD were then captured until June 2018 to ensure all patients would have 5 years of follow-up.

Individuals with incident IBD who had their first intestinal resection during this timeframe (Jan. 1st 2007–June 30th 2018) were then included (total colectomy for UC; any intestinal resection for CD; Table S2) [18]. Further, in order to increase the likelihood that the CD-related resection was to remove areas of ongoing inflammation, we excluded any intestinal resections where the pathology demonstrated dysplasia or colorectal cancer.

### 2.3 | Primary Outcome

Our primary outcome was being ‘treatment free’ within 5 years of initial intestinal resection, defined as the absence of any IBD-related therapy (whether for ongoing inflammation or prophylaxis to prevent recurrence). This included no new prescriptions for IBD-related medications, no unplanned IBD-related hospitalizations, and/or no subsequent IBD-related resections within the 5-year postoperative period. Of note, the first thirty days of postoperative follow-up were excluded to account for corticosteroid tapers as well as immediate post-surgical complications that would be less likely to indicate ongoing disease activity. Further, when considering the need for additional IBD-related surgery among individuals with UC, ICD codes pertaining to completion proctectomies, end ileostomy creation, and ileoanal pouch anastomoses (IPAA) were excluded (Table S2). As a sensitivity analysis, we also included completion proctectomy within our composite outcome, given that in some cases the rectum may remain in situ and require later removal due to ongoing disease activity. IBD-related medications included immunomodulators, advanced therapies (including infusion-based therapies), and all systemic corticosteroid prescriptions, which were obtained from the Prescription Drug Register as well as from the Swedish Quality Register for Inflammatory Bowel Disease (SWIBREG; Table S3) [13, 19]. As has been previously used, IBD-related hospitalizations and surgeries were defined as those in which IBD was the primary associated ICD code [20].

### 2.4 | Additional Covariates

Demographic variables captured included sex, country of birth (Nordic vs. Non-Nordic), and educational level, which were obtained from the Swedish Longitudinal Integrated Database for Health Insurance and Labor Market Studies. IBD-specific variables included age at IBD diagnosis (< 18 years, 18–39 years, 40–59 years and ≥ 60 years), age at intestinal resection, and disease duration prior to resection. Additionally, disease location (Montreal classification), the presence of extraintestinal manifestations of disease, the presence of other immune-mediated diseases (IMIDs), a family history of IBD (first degree relative), and chronic obstructive pulmonary disease (COPD) as a proxy for heavy tobacco use were captured (Tables S4 and S5) [15]. At the time of surgery, the number of advanced therapies previously used, as well as whether immunomodulators had been previously used, were also captured.

### 2.5 | Statistical Analysis

Categorical variables were expressed as a percentage, and continuous variables were expressed as a mean with standard deviation or median with interquartile range (IQR). Given the minimal loss to follow-up and complete longitudinal data available for all residents in Sweden, we conducted univariable and multivariable stepwise logistic regression models to determine the factors associated with being ‘treatment free’ at 5 years. Individual logistic regression models for both UC and CD were conducted to determine adjusted odds ratios (ORs) and resulting 95% confidence intervals (CIs). Time to event (Kaplan–Meier) analysis was also conducted in order to assess the timeframe for needing an IBD-related treatment within the 5 years.

Given recent data suggesting that earlier intestinal resection may be particularly beneficial among older adults, we performed subgroup analyses to determine the factors associated with being ‘treatment free’ at 5 years among adults ≥ 50 years. This age cutoff was chosen both to increase the power of our findings (increase *N* to allow for subgroup analysis), as well as to reflect prior data suggesting a benefit for earlier surgery among adults with IBD ≥ 50 years old [4].

Additionally, as a sensitivity analysis, we introduced a six-month postoperative washout period to account for medications that may be continued or tapered only in the early postoperative phase but not required long-term. This study was approved by the Stockholm Ethics Review Board, and data were analysed using SAS statistical software (version 9.4, SAS Institute Inc., Cary, NC, USA).

## 3 | Results

### 3.1 | Baseline Characteristics

**FIGURE 1 |:**
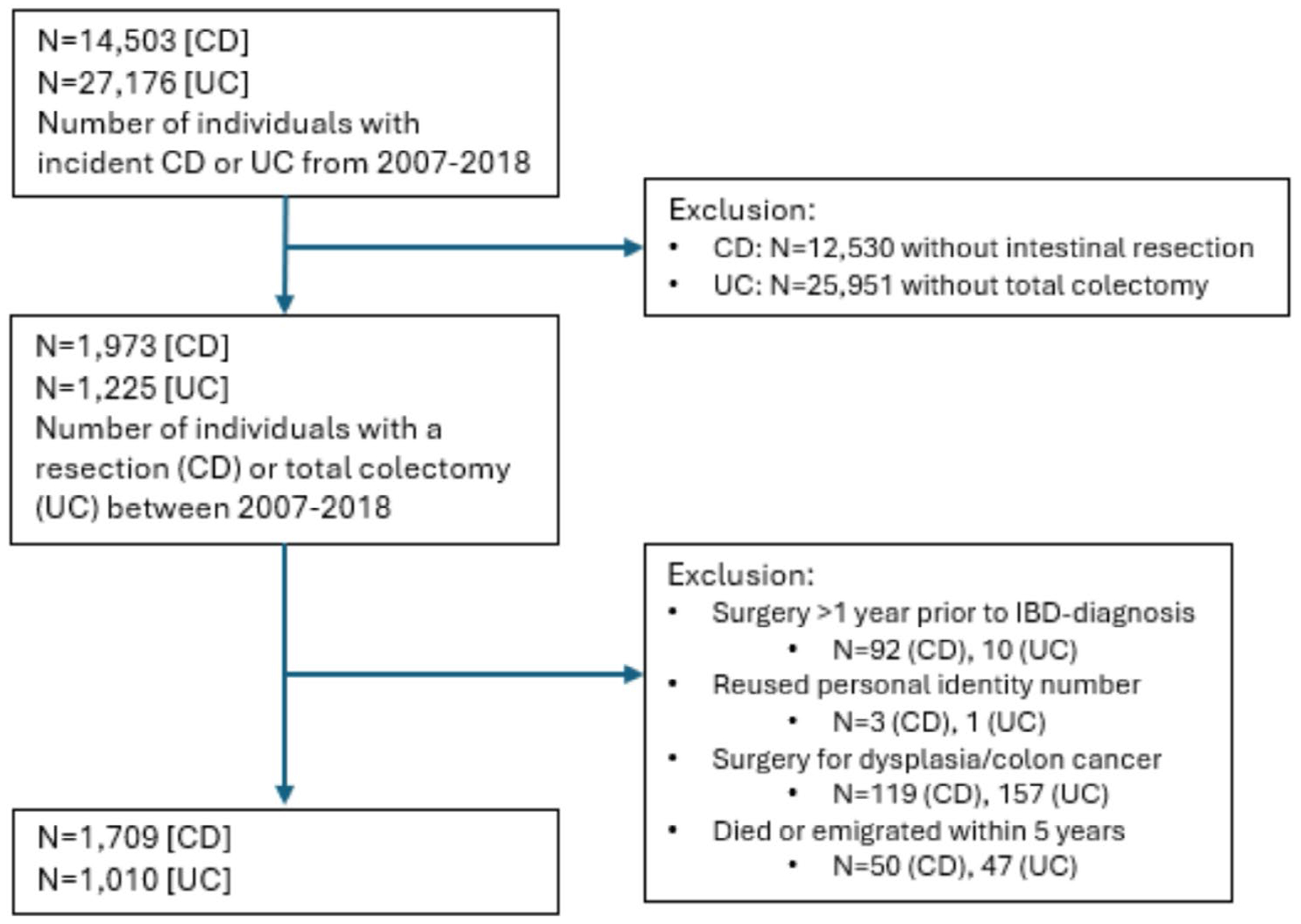
Flow chart.

From 2007 to 2018, there were 41,679 incident cases of IBD, with 2816 patients undergoing their first IBD-related intestinal resection during this period. Notably, 50 individuals with CD and 47 with UC either died or emigrated within 5 years of their initial resection and were excluded from the analysis. This left 1709 individuals with CD who underwent intestinal resection and 1010 with UC who underwent total colectomy, all of whom were followed for at least 5 years (Figure 1). Among those with CD, 48.3% were female, whereas among those with UC, 61.1% were male (Table 1). Overall, the median age at CD resection was 39.2 years (IQR 24.6–58.4) and 38.2 years (IQR 25.2–59.6) among those with UC who underwent colectomy. Disease duration was also short prior to resection, with a median of 0.5 years (IQR 0.0–2.1) for those with CD and 1.2 years (IQR 0.1–2.9) among those with UC.

### 3.2 | Primary Outcome

In total, among those who had an intestinal resection for CD, 341 (21.5%) were ‘treatment free’ at 5 years postop. Of the remaining individuals who required an IBD-related therapy within 5 years postoperatively, approximately 39% were prescribed an immunomodulator, 8% an advanced therapy and 45% a corticosteroid (Table 1). Further, 9.2% required an unplanned IBD-related hospitalisation and 2.3% required a subsequent surgery.

Among those with UC undergoing total colectomy, 262 (42.4%) remained ‘treatment free’ at 5 years postop. Of those who required an IBD-related therapy in the five-year postoperative period, approximately 12% were prescribed an immunomodulator, 5% an advanced therapy and 60% a corticosteroid. Further, 15.8% required an unplanned IBD-related hospitalization and 5.2% required a subsequent IBD-related surgery.

### 3.3 | Factors Associated With Being ‘Treatment Free’ 5-Years Postoperatively

On multivariable analysis of individuals with CD, a longer disease duration prior to surgery (aOR 1.07, 95% CI: 1.00–1.14) and being treatment-naïve to immunomodulators before intestinal resection (aOR 2.39, 95% CI: 1.62–3.55) were associated with higher odds of remaining ‘treatment free.’ Conversely, the presence of COPD, used as a surrogate for heavy tobacco use (aOR 0.21, 95% CI: 0.06–0.69), and the presence of extraintestinal manifestations (aOR 0.64, 95% CI: 0.43–0.97) were associated with lower odds of remaining ‘treatment free’ (Table 2a). In a sensitivity analysis excluding the initial six-month postoperative period, similar factors remained significant (Table S6). Further, when stratifying by disease location, no differences in the need for postoperative treatment were seen.

On multivariable analysis of patients with UC, several factors were associated with being ‘treatment free.’ These included age 40–< 60 years (aOR 1.50, 95% CI: 1.08–2.09), age ≥ 60 years (aOR 1.56, 95% CI: 1.12–2.18), being male (aOR 1.45, 95% CI: 1.11–1.89) and being born in a Nordic country compared to a non-Nordic country (aOR 1.69, 95% CI: 1.09–2.63; Table 2b). In contrast, the presence of extraintestinal manifestations was associated with a lower likelihood of being ‘treatment free’ (aOR 0.48, 95% CI: 0.31–0.73). When excluding the initial six-month postoperative period, age 40 years and older at the time of surgery remained significantly associated with being ‘treatment free’ at 5 years (Table S7). Results were also consistent in a sensitivity analysis that included completion proctectomy as an outcome, with extraintestinal manifestations reducing the odds of being ‘treatment free,’ and older age and Nordic country of birth increasing the odds (Table S8).

### 3.4 | Older Adults: Factors Associated With Being ‘Treatment Free’ 5-Years Postoperatively

Among adults with CD aged 50 years and older, while a longer disease duration increased the odds of being ‘treatment free’ at 5 years, this result was no longer statistically significant (aOR 1.10, 95% CI: 0.99–1.21; Table 3a). However, as observed previously, the presence of COPD and extraintestinal manifestations was significantly associated with a lower odds of being ‘treatment free’ at 5 years.

Among individuals with UC aged 50 years and older, longer disease duration and male sex increased the odds of being ‘treatment free’ at 5 years, though neither remained a significant predictor (Table 3b). Similarly, while extraintestinal manifestations were linked to a lower likelihood of being ‘treatment free,’ this association was not statistically significant.

### 3.5 | Time to IBD-Related Therapy

**FIGURE 2 |:**
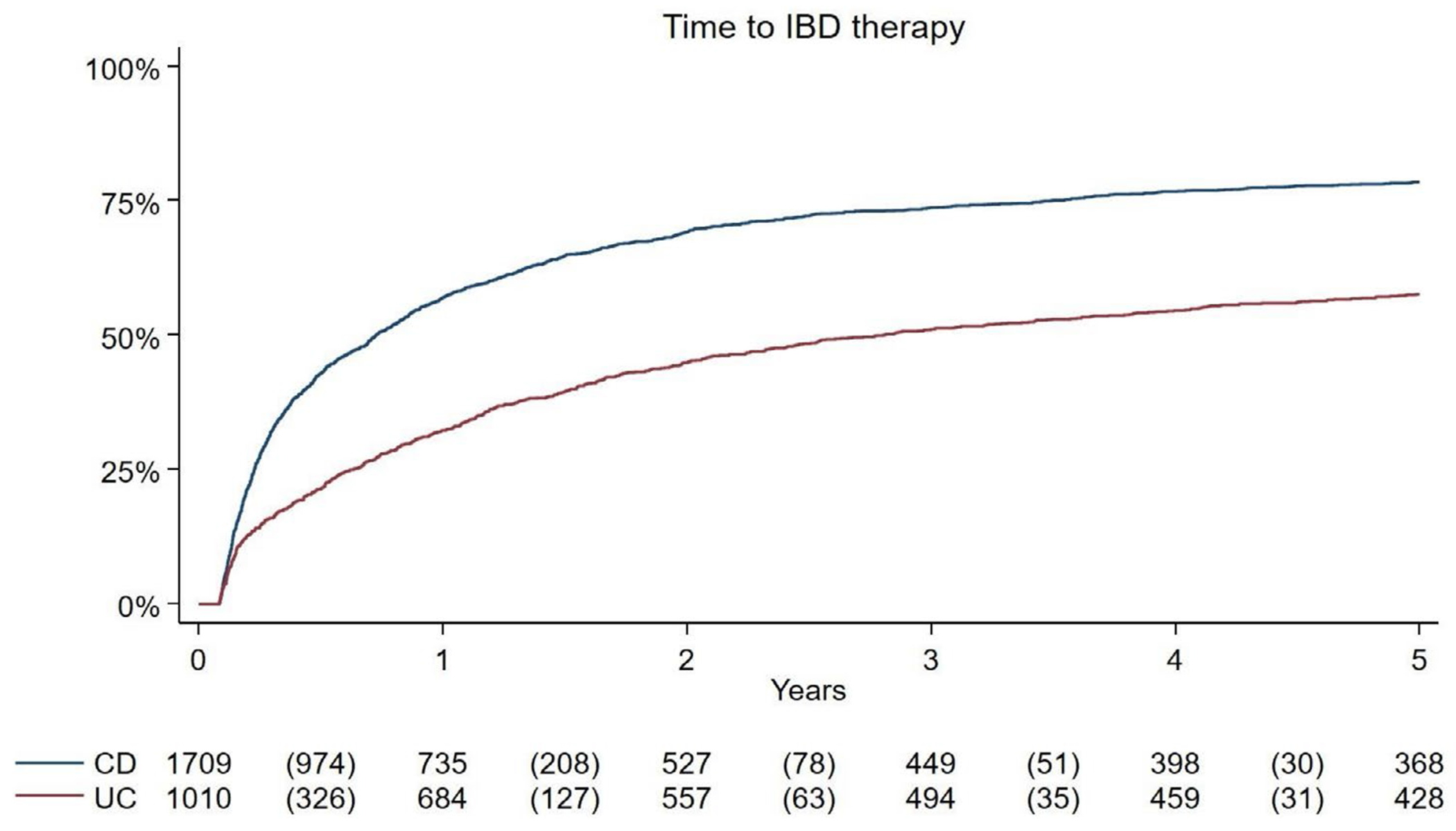
Kaplan–Meier curve showing time to IBD therapy* after initial surgical resection in CD (blue) and UC (red). *IBD therapy: IBD medication (immunomodulators, advanced therapies, corticosteroids), unplanned IBD-related hospitalisation, IBD-related intestinal resection.

On Kaplan–Meier analysis, at 1 year, 57.0% of individuals with CD were prescribed an IBD-related medication or had an unplanned IBD-related hospitalisation or resection; by 2 years, this percentage increased to 69.2%. Among individuals with UC, 32.3% were prescribed an IBD-related medication or had an unplanned IBD-related hospitalisation or resection within 1 year, with this number rising to 44.9% by 2 years (Figure 2). Beyond 2 years, the number of individuals requiring an IBD-related therapy, hospitalisation, or resection plateaued for both UC and CD.

## 4 | Discussion

In this first nationwide study to characterise individuals who may no longer require IBD-related therapy after an initial intestinal resection, we found that 21.5% of individuals with CD and 42.4% of those with UC remained ‘treatment free’ at 5 years. In both UC and CD, the presence of extraintestinal manifestations was linked to a higher likelihood of requiring IBD-related therapy in the 5-year postoperative period. Moreover, among those with CD, a longer disease duration prior to initial surgery and having no prior exposure to an immunomodulator increased the likelihood of remaining ‘treatment free,’ whereas the presence of COPD (surrogate marker for tobacco use) decreased the odds of being ‘treatment free.’ Among those with UC, older age, as well as being male, increased the odds of remaining ‘treatment free’ at 5 years. Notably, the majority of individuals with UC or CD who eventually needed an IBD-related medication, unplanned hospitalization, or surgery did so within two years of their initial resection.

Recent data suggest that earlier intestinal resection in patients with IBD may significantly improve outcomes [4, 21]. Surgery can minimise prolonged inflammation, thereby reducing reliance on corticosteroids, the risks of malnutrition, sepsis, and emergency surgery, while also mitigating functional decline [3, 22]. Further, for select individuals, surgery may eliminate the need for future IBD-related treatments altogether [5]. Despite these findings, limited data characterising these individuals exist.

In our study, among individuals with CD, we found that for every year increase in disease duration prior to initial intestinal resection, an individual had a 7% higher odds of remaining ‘treatment free’ by 5 years. This may suggest that individuals with less severe disease, a more indolent course, may be more likely to achieve sustained remission after CD-related surgery. Supporting this, we found that those who were not previously exposed to immunomodulators, a treatment commonly used as monotherapy for moderate CD in Sweden, were nearly two and a half times more likely to remain ‘treatment free’ at 5 years. These findings also align with prior data showing that a shorter disease duration prior to initial resection is associated with an increased risk of requiring a future CD-related surgical procedure [10]. Additionally, and in accordance with prior data, we found that the presence of COPD among individuals with CD (a surrogate for heavy tobacco use), as well as the presence of extraintestinal manifestations, increased the odds of needing an IBD-related therapy within 5 years [23].

Similarly, among individuals with UC, the presence of extraintestinal manifestations reduced the odds of remaining ‘treatment free’ by > 50%. This finding aligns with existing evidence, as extraintestinal manifestations may portend a more severe disease course or independently necessitate the initiation of an immunosuppressive therapy [24]. Further, we found that older individuals (40–< 60 years and ≥ 60 years) were also 1.5-fold more likely to be ‘treatment free’ at 5 years as compared to individuals 18–39 years of age. While this finding requires further exploration, one plausible explanation is that age-related immunologic and microbiome changes may protect against future pouch-related complications, as has been previously found [25]. Additionally, preference for an end ileostomy over an ileoanal pouch anastomosis in this age cohort may also limit the risk for future pouch-related complications [26]. Although the reasons why males and individuals born in a Nordic country were more likely to remain ‘treatment free’ remain unclear, this may be influenced by genetic and early-life environmental factors, socioeconomic differences, and patient preferences (e.g., those born in Nordic countries are less likely to have an ileoanal pouch or ileorectal anastomosis) [27]. These factors, which shape disease and treatment-related decisions, require further study [28].

While these findings help identify individuals with IBD who are most likely to achieve sustained benefit from surgical intervention, determining this in the older adult population is particularly important. Older adults face unique challenges, including increased vulnerability to medication-related side effects, a higher risk of inflammation-related decline, and a shorter time horizon, making them well-suited for surgical intervention as a potential ‘definitive’ IBD therapy [8, 29]. As such, in our study, we found that older adults with CD who did not have extraintestinal manifestations or COPD were more likely to be ‘treatment-free’ at 5 years. This reinforces the established connection between tobacco use and CD activity, and helps define the subgroup of older adults with CD who may achieve sustained ‘treatment-free’ remission after initial surgical resection [23]. Additionally, although not statistically significant, for every year increase in disease duration prior to initial intestinal resection, the odds of remaining ‘treatment-free’ at 5 years increased by 10% (CD) and 8% (UC) among older adults. Although larger powered studies are needed, this may suggest that older adults with longer disease durations—likely characterised by ongoing moderate inflammation rather than severe, acute flares—may represent an optimal cohort for surgical intervention, as they may be more likely to remain both treatment- and complication-free following surgery. In all, these findings provide valuable insights to help guide surgical decision-making for older adults, particularly in identifying factors that may lead to sustained ‘treatment-free’ outcomes.

Last, our data suggest that if an IBD-related medication, hospitalisation, or surgery is not required within the first two postoperative years, whether for prophylaxis or treatment of disease recurrence, the likelihood of needing treatment in subsequent years decreases. This has direct clinical implications, offering reassurance to both providers and patients who remain in remission at the 2-year postoperative mark.

This study has several notable strengths. First, as a nationwide study conducted in Sweden, we were able to capture a large and representative sample size of individuals with IBD. Further, with the ability to follow all individuals living in Sweden with minimal loss to follow-up, we are able to minimise selection bias, increasing the generalizability of our findings. Another strength lies in the accuracy of case identification. By leveraging validated ICD and histopathology codes, we achieved a high degree of diagnostic and disease location reliability, with 93%–95% accuracy in identifying individuals with IBD [12]. Further, the linkage of data from multiple registries allowed us to examine medication use, hospitalization, and future IBD-related surgeries over a five-year postoperative period. Additionally, although residual confounding exists within all observational studies, we adjusted for several relevant clinical factors known to influence postoperative recurrence in order to limit this possibility. Last, although a high proportion of individuals with UC received corticosteroids postoperatively (possibly reflecting extended corticosteroid tapers and/or the high utilisation of ileorectal anastomoses for UC in Sweden), the robustness of our findings is reinforced by the fact that the characteristics associated with being ‘treatment free’ remained consistent even when excluding IBD-related treatments utilised during the initial 6-month postoperative period [30].

While this study provides valuable insights, certain limitations must be acknowledged. First, although we captured the use of IBD-related medications, it is possible that a proportion of prescriptions were issued for other immune-mediated diseases (IMIDs) rather than for IBD specifically. However, as more than 80% of individuals who required an IBD-related therapy within 5 years did not have another IMID, the likelihood of significant misclassification affecting our primary outcome is low. Second, while we used COPD as a surrogate for current tobacco use as has been done in other registry-based studies, this approach has important limitations; it may both capture former smokers and exclude individuals with lighter smoking histories [15]. Moreover, corticosteroids prescribed for COPD exacerbations could also potentially confound our outcome, although this seems less likely given that COPD significantly reduced the odds of being ‘treatment free’ at 5 years in CD but not in UC. Third, although we excluded individuals who underwent a resection for dysplasia or colorectal cancer, we cannot entirely rule out the possibility that the intestinal resection, particularly among patients with CD, may not have completely resected all areas of active disease. However, our findings showed no significant differences based on disease location, suggesting that this is unlikely to have substantially impacted the results. Fourth, while the study benefits from robust follow-up, the lack of granular clinical details, such as disease activity assessments and patient preferences, limits our ability to determine who was in sustained clinical and/or endoscopic remission off all therapies at 5 years. We also acknowledge that data on infusion-based advanced therapies are incomplete, with one prior study estimating that 77% of infusion data are captured through the National Patient and Prescribed Drug Registers [31, 32]. Nevertheless, if the disease were severe enough to warrant an infusion-based therapy, it is likely that patients would also have required corticosteroids, additional intestinal resection, or hospitalisation within the 5-year period, making it unlikely that this limitation substantially affected our findings.

In summary, this nationwide study is among the first to characterise individuals with IBD who may remain ‘treatment free’ at 5 years following initial IBD-related intestinal resection. Of the 21.5% of individuals with CD and 42.4% with UC achieving this outcome, the absence of extraintestinal manifestations emerged as a consistent predictor of sustained remission. Additional factors associated with remaining ‘treatment free’ included the absence of COPD (a surrogate for tobacco use), longer disease duration before surgery, and no prior exposure to immunomodulators in CD, while older age and male sex were linked to sustained remission in UC. Importantly, these findings invite reflection on current STRIDE and SPIRIT consensus frameworks, which position surgery prevention as a central tenet of disease management [33, 34]. Our results suggest that for carefully selected patients, surgery itself may represent a pathway to durable remission, laying the groundwork for the concept of ‘precision surgery’ within the broader paradigm of precision medicine. Future research should build upon these findings, specifically aiming to refine predictive models that can better identify patients for whom surgery may serve as both a ‘definitive’ and sustained treatment for ongoing IBD-related inflammation.

## Supplementary Material

Supplementary Tables

Additional supporting information can be found online in the Supporting Information section. Table S1: International Classification of Disease (ICD) codes and SNOMED codes defining inflammatory bowel diseases (IBD). Table S2: Colon surgery codes associated with inflammatory bowel disease. Table S3: Medication ATC-codes. Table S4: Definitions and diagnostic codes used to define ulcerative colitis and Crohn’s disease according to the Montreal classification since the start of ICD-10 (1997). Table S5: Diagnostic codes pertaining to an immune-mediated inflammatory disease (IMID) and extraintestinal manifestation of disease (EIMs). Table S6: Univariable and multivariable logistic regression models for the probability of being ‘treatment-free’ at 5 years among individuals with CD (excluding the initial 6-month postoperative period). Table S7: Univariable and multivariable logistic regression models for the probability of being ‘treatment-free’ at 5 years among individuals with UC (excluding the initial 6-month postoperative period). Table S8: Univariable and multivariable logistic regression models for the probability of being ‘treatment-free’ at 5 years among individuals with UC (including completion proctectomy as an outcome).

## Figures and Tables

**TABLE 1 |:** Baseline characteristics of patients with CD or UC who had an IBD-related intestinal resection.

Characteristic	CD			UC		
	No therapy post-surgery	Therapy post-surgery	Total	No therapy post-surgery	Therapy post- surgery	Total
*N*	368	1341	1709	428	582	1010
Sex, *n* (%)						
Female	169 (45.9)	657 (49.0)	826 (48.3)	148 (34.6)	245 (42.1)	393 (38.9)
Male	199 (54.1)	684 (51.0)	883 (51.7)	280 (65.4)	337 (57.9)	617 (61.1)
Age at surgery/start of follow-up (years)						
Median (IQR)	41.4 (25.6–60.8)	38.6 (24.5–57.7)	39.2 (24.6–58.4)	42.0 (26.7–61.6)	36.4 (24.3–57.7)	38.2 (25.2–59.6)
Categories, *n* (%)						
< 18 years	16 (4.3)	96 (7.2)	112 (6.6)	21 (4.9)	40 (6.9)	61 (6.0)
18–< 40 years	160 (43.5)	600 (44.7)	760 (44.5)	180 (42.1)	281 (48.3)	461 (45.6)
40–< 60 years	95 (25.8)	349 (26.0)	444 (26.0)	110 (25.7)	128 (22.0)	238 (23.6)
≥ 60 years	97 (26.4)	296 (22.1)	393 (23.0)	117 (27.3)	133 (22.9)	250 (24.8)
Disease duration until IBD-related surgery						
Median (IQR)	0.3 (−0.0–1.9)	0.5 (0.0–2.2)	0.5 (0.0–2.1)	1.4 (0.2–3.1)	1.0 (0.1–2.8)	1.2 (0.1–2.9)
Categories, *n* (%)						
0–< 1 years	244 (66.3)	817 (60.9)	1061 (62.1)	181 (42.3)	294 (50.5)	475 (47.0)
1–< 5years	88 (23.9)	383 (28.6)	471 (27.6)	198 (46.3)	223 (38.3)	421 (41.7)
5–< 10 years	34 (9.2)	136 (10.1)	170 (9.9)	49 (11.4)	63 (10.8)	112 (11.1)
≥ 10 years	2 (0.5)	5 (0.4)	7 (0.4)	(0.0)	2 (0.3)	2 (0.2)
Year at start of follow-up, *n* (%)						
2007–2011	152 (41.3)	466 (34.8)	618 (36.2)	143 (33.4)	216 (37.1)	359 (35.5)
2012–2014	94 (25.5)	377 (28.1)	471 (27.6)	139 (32.5)	177 (30.4)	316 (31.3)
2015–2018	122 (33.2)	498 (37.1)	620 (36.3)	146 (34.1)	189 (32.5)	335 (33.2)
Education level (years), *n* (%)						
< 9	111 (30.2)	363 (27.1)	474 (27.7)	126 (29.4)	151 (25.9)	277 (27.4)
10–12	167 (45.4)	646 (48.2)	813 (47.6)	202 (47.2)	282 (48.5)	484 (47.9)
> 12	89 (24.2)	324 (24.2)	413 (24.2)	99 (23.1)	148 (25.4)	247 (24.5)
Missing	1 (0.3)	8 (0.6)	9 (0.5)	1 (0.2)	1 (0.2)	2 (0.2)
Country of birth						
Nordic	330 (89.7)	1169 (87.2)	1499 (87.7)	393 (91.8)	511 (87.8)	904 (89.5)
Non-Nordic	38 (10.3)	172 (12.8)	210 (12.3)	35 (8.2)	71 (12.2)	106 (10.5)
Montreal stage CD at start of follow-up, *n* (%)						
L1 (ileal)	125 (34.0)	449 (33.5)	574 (33.6)			
L2 (colonic)	38 (10.3)	151 (11.3)	189 (11.1)			
L3/LX (ileocolonic or location not defined)	195 (53.0)	723 (53.9)	918 (53.7)			
Missing	10 (2.7)	18 (1.3)	28 (1.6)			
Perianal	12 (3.3)	66 (4.9)	78 (4.6)			
Montreal stage UC at start of follow-up, *n* (%)						
E1 (ulcerative proctitis)				12 (2.8)	23 (4.0)	35 (3.5)
E2 (left sided UC)				38 (8.9)	51 (8.8)	89 (8.8)
E3 (extensive UC)				318 (74.3)	407 (69.9)	725 (71.8)
EX (extent not defined)				56 (13.1)	100 (17.2)	156 (15.4)
Missing				4 (0.9)	1 (0.2)	5 (0.5)
Extraintestinal manifestations at start of follow-up, *n* (%)						
Primary sclerosing cholangitis	(0.0)	10 (0.7)	10 (0.6)	8 (1.9)	21 (3.6)	29 (2.9)
Other extraintestinal manifestations	31 (8.4)	176 (13.1)	207 (12.1)	36 (8.4)	84 (14.4)	120 (11.9)
First degree relative with IBD						
Yes	61 (16.6)	239 (17.8)	300 (17.6)	77 (18.0)	124 (21.3)	201 (19.9)
No	307 (83.4)	1102 (82.2)	1409 (82.4)	351 (82.0)	458 (78.7)	809 (80.1)
Number of other IMIDs						
0	317 (86.1)	1091 (81.4)	1408 (82.4)	369 (86.2)	462 (79.4)	831 (82.3)
1	44 (12.0)	215 (16.0)	259 (15.2)	52 (12.1)	95 (16.3)	147 (14.6)
≥ 2	7 (1.9)	35 (2.6)	42 (2.5)	7 (1.6)	25 (4.3)	32 (3.2)
COPD (proxy for tobacco)						
Yes	3 (0.8)	44 (3.3)	47 (2.8)	11 (2.6)	21 (3.6)	32 (3.2)
No	365 (99.2)	1297 (96.7)	1662 (97.2)	417 (97.4)	561 (96.4)	978 (96.8)
Number of prior advanced therapies						
0	308 (83.7)	1028 (76.7)	1336 (78.2)	247 (57.7)	353 (60.7)	600 (59.4)
1	42 (11.4)	210 (15.7)	252 (14.7)	110 (25.7)	159 (27.3)	269 (26.6)
≥ 2	18 (4.9)	103 (7.7)	121 (7.1)	71 (16.6)	70 (12.0)	141 (14.0)
Prior use of IMM						
None	251 (68.2)	673 (50.2)	924 (54.1)	173 (40.4)	258 (44.3)	431 (42.7)
Only IMM	66 (17.9)	398 (29.7)	464 (27.2)	99 (23.1)	145 (24.9)	244 (24.2)
IMM + Advanced therapy	51 (13.9)	270 (20.1)	321 (18.8)	156 (36.4)	179 (30.8)	335 (33.2)
Reason for censoring, *n* (%)						
Outcome event	(0.0)	1341 (100.0)	1341 (78.5)	(0.0)	582 (100.0)	582 (57.6)
Azathioprine	(0.0)	485 (36.2)	485 (28.4)	(0.0)	51 (8.8)	51 (5.0)
6-MP	(0.0)	36 (2.7)	36 (2.1)	(0.0)	15 (2.6)	15 (1.5)
Anti-TNF	(0.0)	91 (6.8)	91 (5.3)	(0.0)	23 (4.0)	23 (2.3)
Vedolizumab	(0.0)	5 (0.4)	5 (0.3)	(0.0)	4 (0.7)	4 (0.4)
Ustekinumab	(0.0)	8 (0.6)	8 (0.5)	(0.0)	(0.0)	(0.0)
Tofacitinib	(0.0)	(0.0)	(0.0)	(0.0)	3 (0.5)	3 (0.3)
Corticosteroid prescription	(0.0)	606 (45.2)	606 (35.5)	(0.0)	346 (59.5)	346 (34.3)
Unplanned IBD-related hospitalisation	(0.0)	124 (9.2)	124 (7.3)	(0.0)	92 (15.8)	92 (9.1)
IBD-related surgery	(0.0)	31 (2.3)	31 (1.8)	(0.0)	30 (5.2)	30 (3.0)
End of follow-up (5 years or Jun 30, 2023)	368 (100.0)	(0.0)	368 (21.5)	428 (100.0)	(0.0)	428 (42.4)

Abbreviations: COPD, Chronic obstructive pulmonary disease; IMID, Immune-mediated disease; IMM, Immunomodulator; OEM, Other extraintestinal manifestations.

**TABLE 2 |:** Univariable and multivariable logistic regression models for the probability of being ‘treatment-free’ at 5 years among individuals with (a) CD, (b) UC.

Variable	Univariate model		Multivariate model	
	OR (95% CI)	*p*	OR (95% CI)	*p*
**(a)**				
Sex				
Female (reference)	1.00	—	Eliminated	—
Male	1.13 (0.90–1.43)	0.30	Eliminated	—
Age at surgery				
< 18 years	0.63 (0.36–1.09)	0.098	Eliminated	—
18–< 40 years (reference)	1.00	—	Eliminated	—
40–< 60 years	1.02 (0.77–1.36)	0.89	Eliminated	—
≥ 60 years	1.23 (0.92–1.64)	0.16	Eliminated	—
Disease duration (continuous)	0.96 (0.91–1.01)	0.14	1.07 (1.00–1.14)	0.043
Year at surgery				
2007–2011 (reference)	1.00	—	Eliminated	—
2012–2014	0.76 (0.57–1.02)	0.070	Eliminated	—
2015–2018	0.75 (0.57–0.98)	0.037	Eliminated	—
Education level (years), *n* (%)				
< 9 (reference)	1.00	—	Eliminated	—
10–12	0.84 (0.64–1.10)	0.21	Eliminated	—
> 12	0.90 (0.65–1.23)	0.51	Eliminated	—
Country of birth				
Non-Nordic (reference)	1.00	—	Eliminated	—
Nordic	1.28 (0.88–1.85)	0.20	Eliminated	—
Montreal stage				
L2 (reference)	1.00	—	Eliminated	—
L1/L3/LX	0.96 (0.68–1.35)	0.82	Eliminated	—
EIM				
PSC				
No (reference)	1.00	—	Eliminated	—
Yes	—	—	Eliminated	—
OEM				
No (reference)	1.00	—	1.00	—
Yes	0.61 (0.41–0.91)	0.015	0.64 (0.43–0.97)	0.033
First degree relative with IBD				
No (reference)	1.00	—	Eliminated	—
Yes	0.92 (0.67–1.25)	0.58	Eliminated	—
Number of other IMIDs				
0	1.45 (0.64–3.30)	0.37	Not included	—
1	1.02 (0.43–2.45)	0.96	Not included	—
≥ 2 (reference)	1.00	—	Not included	—
COPD				
No (reference)	1.00	—	1.00	—
Yes	0.24 (0.07–0.78)	0.018	0.21 (0.06–0.69)	0.010
Number of prior advanced therapies				
0	1.71 (1.02–2.87)	0.041	Eliminated	—
1	1.14 (0.63–2.09)	0.66	Eliminated	—
≥ 2 (reference)	1.00	—	Eliminated	—
Prior use of IMM				
None	1.97 (1.42–2.75)	< 0.001	2.39 (1.62–3.55)	< 0.001
Only IMM	0.88 (0.59–1.31)	0.52	0.95 (0.63–1.43)	0.80
IMM + Advanced therapy (reference)	1.00	—	1.00	—
**(b)**				
Sex				
Female (reference)	1.00	—	1.00	—
Male	1.38 (1.06–1.78)	0.016	1.45 (1.11–1.89)	0.007
Age at surgery				
< 18 years	0.82 (0.47–1.44)	0.49	0.79 (0.44–1.40)	0.41
18–< 40 years (reference)	1.00	—	1.00	—
40–< 60 years	1.34 (0.98–1.84)	0.068	1.50 (1.08–2.09)	0.016
≥ 60 years	1.37 (1.01–1.87)	0.046	1.56 (1.12–2.18)	0.009
Disease duration (continuous)	1.04 (0.98–1.10)	0.22	Eliminated	—
Year at surgery				
2007–2011 (reference)	1.00	—	Eliminated	—
2012–2014	1.19 (0.87–1.61)	0.27	Eliminated	—
2015–2018	1.17 (0.86–1.58)	0.32	Eliminated	—
Education level (years), *n* (%)				
< 9 (reference)	1.00	—	Eliminated	—
10–12	0.86 (0.64–1.16)	0.32	Eliminated	—
> 12	0.80 (0.57–1.13)	0.21	Eliminated	—
Country of birth				
Non-Nordic (reference)	1.00	—	1.00	—
Nordic	1.56 (1.02–2.39)	0.041	1.69 (1.09–2.63)	0.019
Montreal stage				
E1 (reference)	1.00	—	1.00	—
E2	1.43 (0.63–3.22)	0.39	1.42 (0.62–3.28)	0.41
E3	1.50 (0.73–3.06)	0.27	1.74 (0.84–3.62)	0.14
EX	1.14 (0.53–2.45)	0.74	1.26 (0.58–2.75)	0.56
EIM				
PSC				
No (reference)	1.00	—	Eliminated	—
Yes	0.51 (0.22–1.16)	0.11	Eliminated	—
OEM				
No (reference)	1.00	—	1.00	—
Yes	0.54 (0.36–0.82)	0.004	0.48 (0.31–0.73)	< 0.001
First degree relative with IBD				
No (reference)	1.00	—	Eliminated	—
Yes	0.81 (0.59–1.11)	0.19	Eliminated	—
Number of other IMIDs				
0	2.85 (1.22–6.67)	0.016	Not included	—
1	1.95 (0.79–4.83)	0.15	Not included	—
≥ 2 (reference)	1.00	—	Not included	—
COPD				
No (reference)	1.00	—	Eliminated	—
Yes	0.70 (0.34–1.48)	0.35	Eliminated	—
Number of prior advanced therapies				
0	0.69 (0.48–1.00)	0.048	0.97 (0.50–1.88)	0.92
1	0.68 (0.45–1.03)	0.067	0.68 (0.44–1.05)	0.079
≥ 2 (reference)	1.00	—	1.00	—
Prior use of IMM				
None	0.77 (0.58–1.03)	0.075	0.59 (0.34–1.03)	0.062
Only IMM	0.78 (0.56–1.09)	0.15	0.58 (0.30–1.10)	0.095
IMM + Advanced therapy (reference)	1.00	—	1.00	—

Abbreviations: COPD, Chronic obstructive pulmonary disease; EIM, Extraintestinal manifestations; IMID, Immune-mediated disease; IMM, Immunomodulator; OEM, Other extraintestinal manifestations; PSC, Primary sclerosing cholangitis.

**TABLE 3 |:** Univariable and multivariable logistic regression models for the probability of being ‘treatment-free’ at 5 years among individuals ≥ 50 years with (a) CD, (b) UC.

Variable	Univariate model		Multivariate model	
	OR (95% CI)	*p*	OR (95% CI)	*p*
**(a)**				
Sex				
Female (reference)	1.00	—	Eliminated	—
Male	1.03 (0.72–1.49)	0.85	Eliminated	—
Age at surgery				
50–< 60 years	1.00	—	Eliminated	—
60–< 70 years	1.20 (0.79–1.83)	0.40	Eliminated	—
≥ 70 years	0.85 (0.53–1.38)	0.52	Eliminated	—
Disease duration (continuous)	0.99 (0.91–1.08)	0.91	1.10 (0.99–1.21)	0.069
Year at surgery				
2007–2011 (reference)	1.00	—	Eliminated	—
2012–2014	0.65 (0.40–1.03)	0.066	Eliminated	—
2015–2018	0.75 (0.49–1.15)	0.19	Eliminated	—
Education level (years), *n* (%)				
< 9 (reference)	1.00	—	1.00	—
10–12	0.84 (0.55–1.29)	0.44	0.84 (0.54–1.29)	0.42
> 12	1.31 (0.80–2.16)	0.28	1.36 (0.81–2.28)	0.24
Country of birth				
Non-Nordic (reference)	1.00	—	Eliminated	—
Nordic	0.82 (0.41–1.64)	0.58	Eliminated	—
Montreal stage				
L2 (reference)	1.00	—	Eliminated	—
L1/L3/LX	1.09 (0.64–1.84)	0.75	Eliminated	—
EIM				
PSC				
No (reference)	1.00	—	Eliminated	—
Yes	—	—	Eliminated	—
OEM				
No (reference)	1.00	—	1.00	—
Yes	0.47 (0.25–0.87)	0.017	0.50 (0.27–0.95)	0.035
First degree relative with IBD				
No (reference)	1.00	—	Eliminated	—
Yes	1.01 (0.62–1.64)	0.97	Eliminated	—
Number of other IMIDs				
0	2.56 (0.75–8.69)	0.13	Not included	—
1	1.90 (0.52–6.92)	0.33	Not included	—
≥ 2 (reference)	1.00	—	Not included	—
COPD				
No (reference)	1.00	—	1.00	—
Yes	0.21 (0.06–0.69)	0.010	0.19 (0.06–0.64)	0.007
Number of prior advanced therapies				
0	1.40 (0.39–5.00)	0.60	1.51 (0.74–3.10)	0.26
1	1.48 (0.36–6.04)	0.58	0.43 (0.20–0.97)	0.041
≥ 2 (reference)	1.00	—	1.00	—
Prior use of IMM				
None	1.21 (0.64–2.30)	0.56	Eliminated	—
Only IMM	0.41 (0.19–0.89)	0.025	Eliminated	—
IMM + Advanced therapy (reference)	1.00	—	Eliminated	—
**(b)**				
Sex				
Female (reference)	1.00	—	1.00	—
Male	1.45 (0.96–2.19)	0.075	1.44 (0.94–2.19)	0.091
Age at surgery				
50–< 60 years	1.00	—	Eliminated	—
60–< 70 years	1.04 (0.64–1.69)	0.89	Eliminated	—
≥ 70 years	0.99 (0.60–1.64)	0.97	Eliminated	—
Disease duration (continuous)	1.08 (0.98–1.19)	0.10	1.08 (0.98–1.20)	0.13
Year at surgery				
2007–2011 (reference)	1.00	—	Eliminated	—
2012–2014	1.09 (0.67–1.78)	0.73	Eliminated	—
2015–2018	1.07 (0.65–1.75)	0.79	Eliminated	—
Education level (years), *n* (%)				
< 9 (reference)	1.00	—	Eliminated	—
10–12	0.77 (0.48–1.23)	0.28	Eliminated	—
> 12	0.76 (0.43–1.32)	0.32	Eliminated	—
Country of birth				
Non-Nordic (reference)	1.00	—	Eliminated	—
Nordic	1.39 (0.59–3.29)	0.46	Eliminated	—
Montreal stage				
E1 (reference)	1.00	—	1.00	—
E2	3.18 (0.87–11.67)	0.080	3.42 (0.92–12.69)	0.066
E3	2.66 (0.83–8.59)	0.10	2.95 (0.90–9.62)	0.074
EX	1.71 (0.50–5.90)	0.40	2.10 (0.60–7.38)	0.25
EIM				
PSC				
No (reference)	1.00	—	Eliminated	—
Yes	2.30 (0.21–25.57)	0.50	Eliminated	—
OEM				
No (reference)	1.00	—	1.00	—
Yes	0.58 (0.30–1.12)	0.10	0.55 (0.28–1.08)	0.084
First degree relative with IBD				
No (reference)	1.00	—	Eliminated	—
Yes	0.71 (0.40–1.26)	0.24	Eliminated	—
Number of other IMIDs				
0	5.63 (1.61–19.74)	0.007	Not included	—
1	2.85 (0.75–10.82)	0.12	Not included	—
≥ 2 (reference)	1.00	—	Not included	—
COPD				
No (reference)	1.00	—	1.00	—
Yes	0.52 (0.24–1.13)	0.098	0.57 (0.25–1.26)	0.17
Number of prior advanced therapies				
0	0.61 (0.25–1.47)	0.27	Eliminated	—
1	0.52 (0.20–1.35)	0.18	Eliminated	—
≥ 2 (reference)	1.00	—	Eliminated	—
Prior use of IMM				
None	0.77 (0.46–1.31)	0.34	Eliminated	—
Only IMM	0.92 (0.51–1.66)	0.78	Eliminated	—
IMM + Advanced therapy (reference)	1.00	—	Eliminated	—

Abbreviations: COPD, Chronic obstructive pulmonary disease; EIM, Extraintestinal manifestations; IMID, Immune-mediated disease; IMM, Immunomodulator; OEM, Other extraintestinal manifestations; PSC, Primary sclerosing cholangitis.

## Data Availability

The data that support the findings of this study are available on request from the corresponding author. The data are not publicly available due to privacy or ethical restrictions.

## Appendix A

Hans Strid (Department of medicine Solna, Karolinska Institutet, Stockholm, Sweden; Karolinska University Hospital, Stockholm, Sweden), Susanna Jäghult (Department of clinical science and education, Södersjukhuset, Stockholm, Sweden), Caroline Nordenval (Department of Molecular Medicine and Surgery, Karolinska Institutet, Stockholm, Sweden; Department of Colorectal Cancer Karolinska University Hospital, Stockholm, Sweden), Charlotte Hedin (Department of medicine Solna, Karolinska Institutet, Stockholm, Sweden; Karolinska University Hospital, Centre for Digestive Health, Department of Gastroenterology, Dermatovenereology and Rheumatology, Stockholm, Sweden), Olof Grip (Department of Gastroenterology, Skåne University Hospital, Malmö, Sweden), Ulrika L. Fagerberg (Department of Gastroenterology, Sahlgrenska University Hospital, Gothenburg, Sweden) and Karl Marild (Department of Women’s and Children’s Health, Karolinska Institutet, Stockholm, Sweden; Department of Paediatrics, Institute of Clinical Sciences, Sahlgrenska Academy, Gothenburg, Sweden; Department of Paediatric Gastroenterology, Queen Silvia Children’s Hospital, Gothenburg, Sweden).
