# Supplementary material for: Treatment-Free Outcomes Following Surgery for IBD: A Nationwide Cohort Study
Source: Aliment Pharmacol Ther. Author manuscript; Available in PMC 2026 Jan 3. (PMC12757954; doi:10.1111/apt.70432)
Supplement: Supplementary Tables [file NIHMS2127448-supplement-Supplementary_Tables.docx]

**Supplementary Data:**

**Table S1:** *International Classification of Disease* (ICD) codes and SNOMED codes defining inflammatory bowel diseases (IBD)

|  | **ICD-7** | **ICD-8** | **ICD-9** | **ICD-10** | **SNOMED codes*** |
| --- | --- | --- | --- | --- | --- |
| **Swedish National Patient Register** | **1964-1968** | **1969-1986** | **1987-1996** | **1997-** | **1965-** |
| Ulcerative colitis (UC) | 572,20;  572,21;  578,03 | 569,04  563,1; 563,10; 569,02 | 556 | K51 | D6255 *or* M41, M42, M43, M44, M463, or M47 |
| Crohn’s disease (CD) | 572,00;  572,09 | 563,00 | 555 | K50 | D6216 *or* M41, M42, M43, M44, M463, or M47 |
|  |  |  |  |  |  |

*Topographic code T67-T68

**Table S2**: Colon surgery codes associated with inflammatory bowel disease

| **Surgery Codes** | **6^th^ edition**  **1969-1996** | **7^th^ edition (KKÅ97)**  **1997 -** |
| --- | --- | --- |
|  |  |  |
| **Total Colectomy** | 4650-4659 | JFH |
|  |  |  |
| **Resection of the colon +/- small bowel** |  |  |
| Left-sided hemicolectomy | 4640 | JFB43-44 |
| Right-sided hemicolectomy | 4641 | JFB30-31 |
| Ileocecal resection | 4642 | JFB20-21 |
| Resection of the transverse colon | 4643 | JFB40-41 |
| Resection of the colon sigmoid | 4644 | JFB46-47, |
| Other type of partial colon resection | 4649 | JFB50-51 |
| Other type of partial colon or small bowel resection |  | JFB33-34, JFB96-97 |
| Other colon resection with colostomy and distal closure |  | JFB63-64, JGB10-11 |
| Resection of the sigmoid colon with sigmoidostomy and closure of the rectum | 4713 | JFB60-61 |
| Colectomy and ileostomy with closure of the rectum | 4651 | JFH10 |
| Laparoscopic colectomy and ileostomy |  | JFH11 |
| Other colectomy |  | JFH96 |
| Colectomy with ileorectal anastomosis | 4650 | JFH00 |
| Laparoscopic colectomy with ileorectal anastomosis |  | JFH01 |
| Ileorectal anastomosis |  | JFC40 |
| Laparoscopic ileorectal anastomosis |  | JFC41 |
| Colectomy, rectal mucosectomy and ileoanal anastomosis *without* ileostomy |  | JFH30 |
| Colectomy, rectal mucosectomy and ileoanal anastomosis *and* ileostomy |  | JFH33 |
| Proctocolectomy with continent ileostomy, Kock | 4653 | JFH40 |
| Proctocolectomy with ileostomy | 4652 | JFH20 |
|  |  |  |
| **Small bowel/other bowel surgery** |  |  |
| Small bowel resection | 4630-4631 | JFB00-01 |
| Other operation of the small bowel and/or colon | 4660-4668, 4700-4739, 4790-4798 | JFW96 |
| Other laparoscopic operation of the small bowel and/or colon |  | JFW97 |

**Table S3:** Medication ATC-codes

| **Drug category** | **Drug** | **ATC-code** |
| --- | --- | --- |
|  |  |  |
| ***Conventional therapies*** |  |  |
| Immunomodulators | Azathioprine | L04AX01 |
|  | Mercaptopurine | L01BB02 |
|  | Methotrexate | L04AX03/L01BA01 |
|  |  |  |
| ***Biologics*** |  |  |
| Anti-TNF-α | Infliximab | L04AB02 |
|  | Adalimumab | L04AB04 |
|  | Golimumab | L04AB06 |
| Anti- integrins | Vedolizumab | L04AA33 |
| Anti-IL12/23 or anti-IL-23 agents | Ustekinumab | L04AC05 |
|  |  |  |
| ***Small molecules*** |  |  |
| Janus kinase inhibitors | Tofacitinib | L04AA29 |
|  |  |  |
| ***Corticosteroids*** |  |  |
| Corticosteroids, systemic | Betamethasone | H02AB01 |
|  | Dexamethasone | H02AB02 |
|  | Methylprednisolone | H02AB04 |
|  | Prednisolone | H02AB06 |
|  | Prednisone | H02AB07 |
|  | Hydrocortisone | H02AB09 |
|  | Cortisone | H02AB10 |

**Table S4:** Definitions and diagnostic codes used to define ulcerative colitis and Crohn’s disease according to the Montreal classification since the start of ICD-10 (1997).

| **Ulcerative colitis** | **Extent** | **Diagnostic codes** |
| --- | --- | --- |
| E1 | Ulcerative proctitis | K51.2 |
| E2 | Left-sided UC | K51.3; K51.5 |
| E3 or E4 | Extensive UC | K51.0 |
| EX | Extent not defined | K51.4; K51.8; K51.9 |
| **Crohn’s disease** | **Location/Behavior** | **Diagnostic codes** |
| L1 (Location) | Small bowel disease or  terminal ileitis | K50.0 |
| L2 | Colon | K50.1 |
| L3/LX | Ileocecal Crohn’s disease or location not defined | K50.8, K50.9 |
| B1 (Behavior) | Non-stricturing, non-penetrating | None of the ICD-codes for B2 or B3. |
| B2 | Stricturing | Crohn’s disease AND any of the following codes (K56.5; K56.6; K56.7; K62.4) |
| B3 | Penetrating | Crohn’s disease AND any of the following diagnostic codes (K63.0, K63.2, K31.6, N82.3, N82.3, N82.4) OR any of the following surgical procedure codes (JFA76, JFA86). |
| P | Perianal disease modifier | Crohn’s disease AND any of the following diagnostic codes: (K60.3, K60.4, K60.5, K61.0, K61.1, K61.2, K61.3, K61.4, K62.4) OR any of the following surgical procedure codes: (JHD20, JHD30, JHD33, JHD50, JHD60, JHD63, JHA00, JHA20, JHW96) |

**Table S5:** Diagnostic codes pertaining to an immune-mediated inflammatory disease (IMID) and extraintestinal manifestation of disease (EIMs)

| **IMID:** | |  |
| --- | --- | --- |
|  | Multiple sclerosis | ICD-10: G35 |
|  | Psoriasis | ICD-10: L40.0-4, L40.8-9 |
|  | Psoriatic arthritis | ICD-10: L40.5, M07.0-3 |
|  | Rheumatoid arthritis | ICD-10: M05, M06.0, M06.8, M06.9 |
|  | Ankylosing spondylitis | ICD-10: M45 |
|  | Systemic lupus erythematosus | ICD-10: M32.1, M32.8, M32.9 |
|  | Celiac disease/dermatitis herpetiformis | ICD-10: K90.0, L13.0 |
|  | Type 1 diabetes | ICD-10: E10 |
|  | Asthma | ICD-10: J45, J46 |
|  | Sarcoidosis | ICD-10: D86 |
|  | Graves’ disease | ICD-10: E05.0 |
|  | Iridocyclitis | ICD-10: H20 (except H20.2) |
|  | Hashimoto’s hypothyroidism | ICD-10: E06.3 |
|  |  |  |
|  | **EIM:** |  |
|  | Primary sclerosing cholangitis | ICD-10: K830 |
|  | Pyoderma gangrenosum | ICD-10: L88 |
|  | Erythema nodosum | ICD-10: L52 |
|  | Sweet Syndrome | ICD-10: L982 |
|  | Iridocyclitis | ICD-10: H20 |
|  | Arthropathy | ICD-10: M074-M076, M460, M461, M468, M469, M139, M255, M091, M092 |

**Table S6:** Univariable and multivariable logistic regression models for the probability of being ‘treatment-free’ at five years among individuals with CD (excluding the initial 6-month postoperative period)

| **Variable** | **Univariate model** | | **Multivariate model** | |
| --- | --- | --- | --- | --- |
|  | **OR (95%CI)** | **p-value** | **OR (95%CI)** | **p-value** |
| Sex |  |  |  |  |
| Female (reference) | 1.00 | - | 1.00 | - |
| Male | 1.14 (0.90-1.43) | 0.28 | 1.21 (0.95-1.54) | 0.12 |
| Age at surgery |  |  |  |  |
| <18y | 0.47 (0.26-0.86) | 0.015 | 0.48 (0.26-0.90) | 0.021 |
| 18-<40y (reference) | 1.00 | - | 1.00 | - |
| 40-<60y | 1.07 (0.81-1.41) | 0.65 | 0.86 (0.64-1.16) | 0.33 |
| ≥60y | 1.23 (0.92-1.64) | 0.16 | 0.94 (0.68-1.28) | 0.68 |
| Disease duration (continuous) | 0.90 (0.85-0.96) | <0.001 | 1.05 (0.98-1.12) | 0.17 |
| Year at surgery |  |  |  |  |
| 2007-2011 (reference) | 1.00 | - | *Eliminated* | *-* |
| 2012-2014 | 0.77 (0.58-1.02) | 0.073 | *Eliminated* | *-* |
| 2015-2018 | 0.62 (0.47-0.82) | <0.001 | *Eliminated* | *-* |
| Education level (years), n (%) |  |  |  |  |
| <9 (reference) | 1.00 | - | *Eliminated* | *-* |
| 10-12 | 0.95 (0.72-1.25) | 0.71 | *Eliminated* | *-* |
| >12 | 0.97 (0.71-1.33) | 0.86 | *Eliminated* | *-* |
| Country of birth |  |  |  |  |
| Non-Nordic (reference) | 1.00 | - | *Eliminated* | *-* |
| Nordic | 1.13 (0.79-1.60) | 0.51 | *Eliminated* | *-* |
| Montreal stage |  |  |  |  |
| L2 (reference) | 1.00 | - | *Eliminated* | *-* |
| L1/L3/LX | 1.08 (0.77-1.54) | 0.65 | *Eliminated* | *-* |
| EIM |  |  |  |  |
| PSC |  |  |  |  |
| No (reference) | 1.00 | - | *Eliminated* | *-* |
| Yes | - | - | *Eliminated* | *-* |
| OEM |  |  |  |  |
| No (reference) | 1.00 | - | *Eliminated* | *-* |
| Yes | 0.70 (0.48-1.02) | 0.066 | *Eliminated* | *-* |
| First degree relative with IBD |  |  |  |  |
| No (reference) | 1.00 | - | *Eliminated* | *-* |
| Yes | 1.03 (0.77-1.39) | 0.83 | *Eliminated* | *-* |
| Number of other IMIDs |  |  |  |  |
| 0 | 1.77 (0.74-4.25) | 0.20 | *Not included* | *-* |
| 1 | 1.19 (0.47-3.01) | 0.71 | *Not included* | *-* |
| ≥2 (reference) | 1.00 | - | *Not included* | *-* |
| COPD |  |  |  |  |
| No (reference) | 1.00 | - | 1.00 | - |
| Yes | 0.32 (0.11-0.89) | 0.030 | 0.23 (0.08-0.67) | 0.007 |
| Number of prior advanced therapies |  |  |  |  |
| 0 | 2.90 (1.65-5.12) | <0.001 | 3.10 (1.27-7.55) | 0.013 |
| 1 | 1.62 (0.85-3.09) | 0.14 | 1.63 (0.84-3.14) | 0.15 |
| ≥2 (reference) | 1.00 | - | 1.00 | - |
| Prior use of IMM |  |  |  |  |
| None | 3.73 (2.63-5.27) | <0.001 | 2.07 (0.99-4.36) | 0.054 |
| Only IMM | 0.98 (0.66-1.46) | 0.92 | 0.48 (0.22-1.06) | 0.068 |
| IMM + Advanced therapy (reference) | 1.00 | - | 1.00 | - |

EIM: Extraintestinal manifestations;

PSC: Primary sclerosing cholangitis;

OEM: Other extraintestinal manifestations

**Table S7:** Univariable and multivariable logistic regression models for the probability of being ‘treatment-free’ at five years among individuals with UC (excluding the initial 6-month postoperative period)

| **Variable** | **Univariate model** | | **Multivariate model** | |
| --- | --- | --- | --- | --- |
|  | **OR (95%CI)** | **p-value** | **OR (95%CI)** | **p-value** |
| Sex |  |  |  |  |
| Female (reference) | 1.00 | - | 1.00 | - |
| Male | 1.18 (0.89-1.58) | 0.25 | 1.29 (0.96-1.75) | 0.094 |
| Age at surgery |  |  |  |  |
| <18y | 0.71 (0.35-1.44) | 0.34 | 0.54 (0.26-1.14) | 0.11 |
| 18-<40y (reference) | 1.00 | - | 1.00 | - |
| 40-<60y | 1.41 (0.98-2.01) | 0.062 | 1.50 (1.04-2.17) | 0.031 |
| ≥60y | 2.58 (1.84-3.62) | <0.001 | 2.49 (1.72-3.61) | <0.001 |
| Disease duration (continuous) | 1.02 (0.96-1.09) | 0.43 | 1.06 (0.98-1.14) | 0.15 |
| Year at surgery |  |  |  |  |
| 2007-2011 (reference) | 1.00 | - | *Eliminated* | *-* |
| 2012-2014 | 1.29 (0.92-1.81) | 0.13 | *Eliminated* | *-* |
| 2015-2018 | 1.16 (0.83-1.63) | 0.38 | *Eliminated* | *-* |
| Education level (years), n (%) |  |  |  |  |
| <9 (reference) | 1.00 | - | 1.00 | - |
| 10-12 | 0.71 (0.51-0.98) | 0.037 | 0.66 (0.46-0.95) | 0.024 |
| >12 | 0.78 (0.54-1.14) | 0.20 | 0.78 (0.52-1.17) | 0.23 |
| Country of birth |  |  |  |  |
| Non-Nordic (reference) | 1.00 | - | 1.00 | - |
| Nordic | 2.95 (1.62-5.37) | <0.001 | 2.90 (1.57-5.37) | <0.001 |
| Montreal stage |  |  |  |  |
| E1 (reference) | 1.00 | - | *Eliminated* | *-* |
| E2 | 1.44 (0.55-3.73) | 0.46 | *Eliminated* | *-* |
| E3 | 1.61 (0.69-3.74) | 0.27 | *Eliminated* | *-* |
| EX | 1.56 (0.64-3.84) | 0.33 | *Eliminated* | *-* |
| EIM |  |  |  |  |
| PSC |  |  |  |  |
| No (reference) | 1.00 | - | *Eliminated* | *-* |
| Yes | 0.69 (0.28-1.72) | 0.43 | *Eliminated* | *-* |
| OEM |  |  |  |  |
| No (reference) | 1.00 | - | 1.00 | - |
| Yes | 0.69 (0.43-1.08) | 0.11 | 0.63 (0.39-1.01) | 0.056 |
| First degree relative with IBD |  |  |  |  |
| No (reference) | 1.00 | - | *Eliminated* | *-* |
| Yes | 0.98 (0.70-1.39) | 0.93 | *Eliminated* | *-* |
| Number of other IMIDs |  |  |  |  |
| 0 | 2.10 (0.80-5.55) | 0.13 | *Not included* | *-* |
| 1 | 1.90 (0.68-5.28) | 0.22 | *Not included* | *-* |
| ≥2 (reference) | 1.00 | - | *Not included* | *-* |
| COPD |  |  |  |  |
| No (reference) | 1.00 | - | 1.00 | - |
| Yes | 0.89 (0.39-2.01) | 0.77 | 0.47 (0.20-1.11) | 0.085 |
| Number of prior advanced therapies |  |  |  |  |
| 0 | 1.15 (0.76-1.72) | 0.51 | 2.21 (0.92-5.32) | 0.076 |
| 1 | 0.70 (0.44-1.12) | 0.14 | 0.72 (0.43-1.19) | 0.20 |
| ≥2 (reference) | 1.00 | - | 1.00 | - |
| Prior use of IMM |  |  |  |  |
| None | 1.16 (0.84-1.60) | 0.36 | 0.44 (0.20-0.97) | 0.042 |
| Only IMM | 1.13 (0.78-1.63) | 0.52 | 0.37 (0.16-0.87) | 0.023 |
| IMM + Advanced therapy (reference) | 1.00 | - | 1.00 | - |

EIM: Extraintestinal manifestations;

PSC: Primary sclerosing cholangitis;

OEM: Other extraintestinal manifestations

**Table S8** Univariable and multivariable logistic regression models for the probability of being ‘treatment-free’ at five years among individuals with UC (including completion proctectomy as an outcome)

| **Variable** | **N cases (%)** | **Univariate model** | | **Multivariate model** | |
| --- | --- | --- | --- | --- | --- |
|  |  | **OR (95%CI)** | **p-value** | **OR (95%CI)** | **p-value** |
| Sex |  |  |  |  |  |
| Female (reference) | 115 (29.3) | 1.00 | - | 1.00 | - |
| Male | 202 (32.6) | 1.17 (0.89-1.54) | 0.26 | 1.24 (0.94-1.65) | 0.13 |
| Age at surgery |  |  |  |  |  |
| <18y | 18 (29.5) | 1.13 (0.63-2.03) | 0.69 | 1.09 (0.60-1.98) | 0.77 |
| 18-<40y (reference) | 125 (27.1) | 1.00 | - | 1.00 | - |
| 40-<60y | 75 (31.5) | 1.24 (0.88-1.75) | 0.22 | 1.37 (0.96-1.95) | 0.081 |
| ≥60y | 99 (39.4) | 1.76 (1.27-2.43) | <0.001 | 2.00 (1.40-2.85) | <0.001 |
| Disease duration (continuous) |  | 1.04 (0.98-1.10) | 0.25 | *Eliminated* | *-* |
| Year at surgery |  |  |  |  |  |
| 2007-2011 (reference) | 102 (28.4) | 1.00 | - | *Eliminated* | *-* |
| 2012-2014 | 103 (32.5) | 1.21 (0.87-1.68) | 0.25 | *Eliminated* | *-* |
| 2015-2018 | 112 (33.3) | 1.26 (0.91-1.74) | 0.16 | *Eliminated* | *-* |
| Education level (years), n (%) |  |  |  |  |  |
| <9 (reference) | 91 (32.7) | 1.00 | - | *Eliminated* | *-* |
| 10-12 | 144 (29.6) | 0.87 (0.63-1.19) | 0.37 | *Eliminated* | *-* |
| >12 | 82 (33.1) | 1.02 (0.71-1.46) | 0.94 | *Eliminated* | *-* |
| Country of birth |  |  |  |  |  |
| Non-Nordic (reference) | 19 (17.9) | 1.00 | - | 1.00 | - |
| Nordic | 298 (32.9) | 2.24 (1.34-3.76) | 0.002 | 2.21 (1.30-3.75) | 0.003 |
| Montreal stage |  |  |  |  |  |
| E1 (reference) | 8 (22.9) | 1.00 | - | 1.00 | - |
| E2 | 26 (29.2) | 1.39 (0.56-3.46) | 0.48 | 1.45 (0.57-3.68) | 0.43 |
| E3 | 239 (32.9) | 1.65 (0.74-3.69) | 0.22 | 1.98 (0.87-4.49) | 0.10 |
| EX | 44 (27.3) | 1.27 (0.54-3.00) | 0.59 | 1.42 (0.59-3.39) | 0.44 |
| EIM |  |  |  |  |  |
| PSC |  |  |  |  |  |
| No (reference) | 311 (31.6) | 1.00 | - | *Eliminated* | *-* |
| Yes | 6 (20.7) | 0.56 (0.23-1.40) | 0.22 | *Eliminated* | *-* |
| OEM |  |  |  |  |  |
| No (reference) | 289 (32.4) | 1.00 | - | 1.00 | - |
| Yes | 28 (23.3) | 0.64 (0.41-0.99) | 0.046 | 0.58 (0.37-0.92) | 0.020 |
| First degree relative with IBD |  |  |  |  |  |
| No (reference) | 261 (32.2) | 1.00 | - | *Eliminated* | *-* |
| Yes | 56 (27.7) | 0.81 (0.57-1.14) | 0.22 | *Eliminated* | *-* |
| Number of other IMIDs |  |  |  |  |  |
| 0 | 271 (32.6) | 2.09 (0.85-5.15) | 0.11 | *Not included* | *-* |
| 1 | 40 (27.0) | 1.60 (0.62-4.19) | 0.33 | *Not included* | *-* |
| ≥2 (reference) | 6 (18.8) | 1.00 | - | *Not included* | *-* |
| COPD |  |  |  |  |  |
| No (reference) | 310 (31.6) | 1.00 | - | 1.00 | - |
| Yes | 7 (21.9) | 0.61 (0.26-1.41) | 0.25 | 0.44 (0.18-1.06) | 0.066 |
| Number of prior advanced therapies |  |  |  |  |  |
| 0 | 194 (32.3) | 0.85 (0.58-1.25) | 0.41 | 0.71 (0.47-1.07) | 0.099 |
| 1 | 72 (26.8) | 0.65 (0.42-1.01) | 0.055 | 0.58 (0.37-0.91) | 0.018 |
| ≥2 (reference) | 51 (35.9) | 1.00 | - | 1.00 | - |
| Prior use of IMM |  |  |  |  |  |
| None | 135 (31.3) | 0.99 (0.73-1.34) | 0.93 | *Eliminated* | *-* |
| Only IMM | 76 (31.1) | 0.98 (0.69-1.40) | 0.92 | *Eliminated* | *-* |
| IMM + Advanced therapy (reference) | 106 (31.5) | 1.00 | - | *Eliminated* | *-* |

EIM: Extraintestinal manifestations;

PSC: Primary sclerosing cholangitis;

OEM: Other extraintestinal manifestations
